# Metabolic changes of thalamus assessed by ^1^H-MRS spectroscopy in patients of cervical spondylotic myelopathy following decompression surgery

**DOI:** 10.3389/fneur.2024.1513896

**Published:** 2025-01-08

**Authors:** Jiangqin Zheng, Yujin Zhang, Baogen Zhao, Ning Wang, Ting Gao, Li Zhang

**Affiliations:** ^1^Department of Radiology, Hainan Maternal and Child Health Centre, Haikou, China; ^2^Department of Radiology and Nuclear Medicine, The First Hospital of Hebei Medical University, Shijiazhuang, China

**Keywords:** cervical spondylotic myelopathy, magnetic resonance spectroscopy, thalamus, modified Japanese orthopedic association, postoperative

## Abstract

**Objective:**

To assess the changes of thalamic metabolites before and after surgery in patients with Cervical Spondylotic Myelopathy (CSM) using Hydrogen Proton Magnetic Resonance Spectroscopy (^1^H-MRS) and to investigate its association with improvement in neurological function.

**Methods:**

Forty-eight CSM patients who underwent cervical decompression surgery from December 2022 to June 2023 were included, and 33 healthy volunteers were recruited. All subjects underwent bilateral thalamic ^1^H-MRS scans before the surgical procedure, and subsequently again 6 months later. Neurological function was assessed pre-operatively and post-operatively (6 months) in all patients with CSM using the modified Japanese Orthopedic Association (mJOA). The changes of mJOA (ΔmJOA = postoperative mJOA–preoperative mJOA) were employed as an indicator of neurological improvement. The pre- and postoperative metabolic ratio of *N*-acetylaspartate/creatine (NAA/Cr), choline/creatine (Cho/Cr), myo-inositol/creatine (mI/Cr), glutamate and glutamine complex/creatine (Glx/Cr) were statistically compared in CSM patients and healthy controls (HCs). A correlation analysis was conducted to determine the relationship between alterations in pre- and postoperative metabolite ratios (ΔNAA/Cr, ΔCho/Cr, ΔmI/Cr, ΔGlx/Cr) and ΔmJOA.

**Results:**

Compared to the HCs, patients with CSM showed significantly lower pre- and post-operative NAA/Cr (*Z* = −4.235, *p* < 0.001; *Z* = −3.184, *p* = 0.001), Cho/Cr (*Z* = −5.050, *p* < 0.001; (*Z* = −2.624, *p* = 0.007) and mI/Cr (*Z* = −3.739, *p* = 0.001; *Z* = −2.014, *p* = 0.044). There was no difference in Glx/Cr between patients in patients with CSM, either preoperatively or postoperatively, compared to HCs. Post-operative NAA/Cr (*Z*  = −2.285, *p* = 0.041) and mI/Cr (*Z* = −2.925, *p* = 0.021) were increased in CSM patients compared to pre-operative NAA/Cr and mI/Cr. In CSM patients, ΔmI/Cr correlated significantly with ΔmJOA (*r* = 0.507, *p* < 0.001).

**Conclusion:**

The preliminary findings indicate that metabolites in the thalamus of CSM patients exhibit changes following surgery. Additionally, it has been demonstrated that elevated postoperative mI correlates with improvements in neurological function.

## Introduction

Cervical spondylotic myelopathy (CSM) represents a prevalent form of degenerative cervical spondylosis. The primary underlying cause of this condition is the compression of the spinal cord, which may result from the presence of herniated discs, osteophytes, and other factors (1). CSM has an insidious onset in the early stages of the disease, manifesting as neck pain and stiffness. In the later stages of the disease, neurological impairments may become apparent, including motor and sensory dysfunction, as well as paralysis and disorders of micturation (2).

The conservative treatment of mild CSM is the general approach. Conversely, surgical decompression of the spinal cord is the recommended course of action for patients with advanced severe disease who present with intractable pain and progressive neurological deterioration (3). Following surgical decompression of the spinal cord, patients typically demonstrate an initial improvement in motor function and gait, with subsequent, unpredictable improvements in sensory function (4). A study has demonstrated that patients who have undergone CSM surgery are at an increased risk of progressive deterioration in sensory function in the postoperative period (5). This suggests that the recovery of motor and sensory function subsequent to surgical intervention cannot be evaluated exclusively the degree of injury to the spinal cord component. The presumed reason for this is that impairment of sensory function in patients with CSM cannot be measured solely on the basis of the degree of local spinal cord injury.

Moreover, evidence suggests that chronic damage resulting from spinal cord compression has the potential to cause retrograde brain damage (6). The existing body of research confirming retrograde brain damage and functional remodeling in CSM patients has focused on the motor centers of the cerebral cortex (7). This provides a plausible explanation for the neurological remodeling observed in postoperative CSM patients. In a study by List et al. utilizing diffusion tensor imaging (DTI) to examine structural alterations of white matter in patients with purely hereditary spastic paraplegia (HSP), it was observed that the fractional anisotropy (FA) values of the internal capsule and corpus callosum were markedly reduced in patients with HSP in comparison to the control group (8). This finding suggests the possibility of retrograde brain damage. Erschbamer et al. also identified alterations in brain metabolites through the utilization of ^1^H-MRS in a rat model of experimental spinal cord injury (SCI) (9).

The study conducted by Wang L et al. revealed that CSM results in sensory-motor cortical atrophy (10); however, the underlying mechanisms responsible for the observed delay in postoperative sensory remodeling remain unclear. The thalamus plays a pivotal role in the transduction of sensory information within the human body, facilitating the projection of sensory impulses to the cerebral cortex. During the bilateral finger-tappingtask, Bernabéu-Sanz Á et al. found in CS patients an increased activation in the cerebellum and basal ganglia (11). The cerebellum processes the afferent inputs from the spinal cord, whereas the thalamus is a relay nucleus to the motor cortex with internal loops from the basal ganglia and the cerebellum. It has been put forth that the thalamus plays a role in the reconstruction and recovery of sensory functions (12). However, there is a dearth of research examining the alterations in the thalamus and the restoration of sensory function prior to and following decompression surgery in patients with CSM.

NAA is an abundant neurotransmitter found exclusively in neurons (13). A decrease in NAA concentration indicates an impairment of neuronal function, reflecting mitochondrial dysfunction and decreased neuronal density (14). Cr is a central molecule in metabolism, which is found in particularly high concentrations in tissues with high energy requirements, e.g., the central nervous system. Choline is a marker of membrane turnover and glia (15), so Cho/Cr may be increased as cell membranes are being disrupted. MI is thought to be a marker of glial mass with differing levels in different diseases (16). Glutamine, a neutral amino acid, is normally a nontoxic ammonia carrier in the CNS and its synthesis represents an astrocyte protection to neurons during increases of blood ammonia concentrations (17).

The objective of this study was to examine the alterations in thalamic metabolites in CSM patients prior to and following surgical intervention through the utilization of hydrogen proton magnetic resonance spectroscopy (^1^H-MRS). Moreover, the study aimed to determine whether there is a correlation between these metabolic alterations and the improvement of neurological function. This would provide a theoretical basis for understanding how cerebral function is remodeled in CSM patients following spinal cord injury.

## Materials and methods

### Participants

A total of 61 patients diagnosed with CSM were treated between December 2022 and June 2023. To eliminate the potential confounding effects of different dominant cerebral hemispheres, all subjects selected to participate in this study were required to be right-handed. Of the total number of patients, 54 met the pre-specified inclusion criteria and were invited to participate in the study. Ultimately, 48 patients elected to take part in the study (34 males and 14 females, aged 31–70 years, with a mean age of 54.88 ± 10.84 years). The mean duration of symptoms from disease onset to the data of the MRI examination was 9.10 ± 4.83 months (range, 1 month-20 months). In order to be eligible for inclusion in the study, the following criteria had to be met: (a) The magnetic resonance imaging (MRI) scan had to demonstrate evidence of compression of the cervical spinal cord, including hypertrophy of the ligamentum flavum, cervical spondylosis, and disc herniation. (b) The patient displayed clinical indications of a cervical spinal cord injury. (c) No evidence was found to suggest the presence of amyotrophic lateral sclerosis, an intramedullary tumor, or peripheral neuropathy. (d) No contraindications to MRI examination were identified. (e) The subject had consented to participate in the study of their own volition. The following criteria will result in exclusion from the study: (a) There is no requirement for surgical intervention. (b) A history of depressive, anxiety-related, substance-related, or other psychiatric disorders. (c) A history of psychiatric or neurological disorders among immediate family members. (d) The MRI findings were consistent with the presence of cerebral anomalies, including tumors, hemorrhages, or infarcts. (e) The patient presents with a traumatic injury to the spinal cord. (f) The presence of contraindications to MRI examination was identified. A total of 33 healthy controls were also recruited for the study (18 males and 15 females, aged between 24 and 72 years, with a mean age of 51.79 ± 12.94 years).

The study was conducted in accordance with the ethical principles set forth in the Declaration of Helsinki and received approval from the Medical Ethics Committee of the institution. Prior to their participation, written informed consent was obtained from all volunteers and patients.

### Neurological function evaluation

A modified Japanese Orthopedic Association (mJOA) score was employed to evaluate all subjects’ motor function prior to undergoing magnetic resonance imaging, both preoperatively and at the 6-month postoperative follow-up. The assessment encompassed an evaluation of motor function in the upper and lower extremities, sensory deficits affecting the extremities and the trunk, and bladder function (18). A maximum score of 18 indicated normal motor function, whereas lower scores indicated more severe dysfunction. The changes of mJOA (△mJOA = postoperative mJOA–preoperative mJOA) was used as an indicator of neurological improvement.

### MR imaging and MR spectroscopy

All subjects underwent preoperative and six-month postoperative ^1^H-MRS scans in the initial examination on an early morning MRI machine. MRI was conducted using a Philips Ingenia-CX 3.0 T MRI system with a 32-channel dedicated head coil. A cranial FSE sequence 3D-T2WI anatomical image scan was initially conducted, employing the following parameters: TR = 2,500 ms, TE = 248 ms, flip angle = 90°, matrix = 252 × 252, FOV = 250 × 250 mm^2^, resolution = 1 mm × 1 mm × 1 mm, slice thickness = 1 mm, slice spacing = 0 mm, scan time = 4min28s.

**Figure 1:**
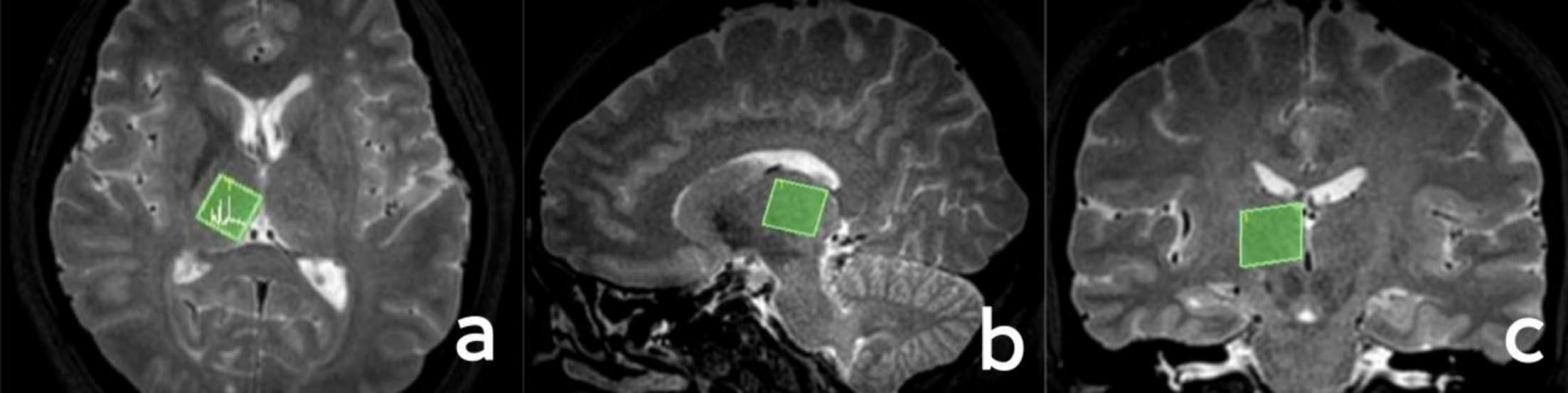
Localization of the thalamus. **(A)** Axial position; **(B)** sagittal position; **(C)** coronal position.

In accordance with the T2WI anatomical localization map, a 15 × 15 × 15 mm voxel-sized region of interest was manually positioned in the left thalamus (it is imperative that the cerebrospinal fluid is avoided as much as possible when placing it), and the region of interest in the right thalamus should be symmetrical with the left side when scanning the right thalamus. A single voxel water suppression point-resolved spectroscopy/sequence (PRESS) was employed for the ^1^H-MRS scanning, with the following parameters: TR = 2000 ms, TE = 38 ms, NSA = 128, bandwidth = 2000 Hz, and scan time 5min14s. Prior to scanning, both manual and automatic high-order homogenization were conducted, and automatic water suppression was performed with a water peak half-height linewidth (FWHM) < 15. The regions of interest for the localization of the thalamus are illustrated in Figure 1.

### Processing of MR imaging data

**Figure 2:**
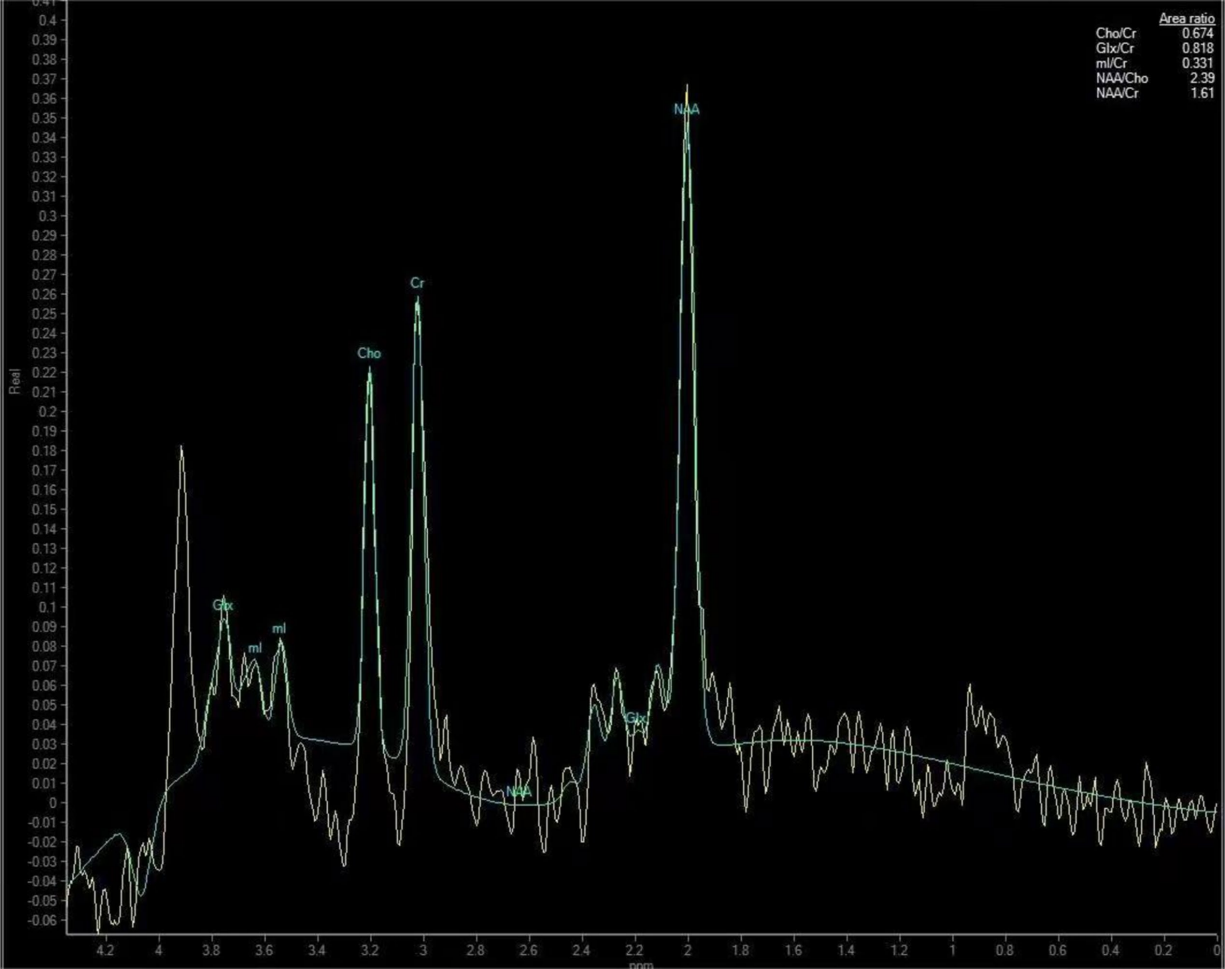
Thalamic metabolite spectra of a 50-year-old male patient with CSM. The patient’s neck pain had lasted for 10 months and the numbness in his hands had lasted for about 6 months.

The data obtained from the scan were transferred to a Philips IntelliSpace Portal 9.0 post-processing workstation, where spectral line plotting was performed using the inbuilt spectral analysis software (Spectroview) of the aforementioned workstation. Thalamus was initially identified as the optimal anatomical location for spectral line fitting. Subsequently, a short TE editing script was selected for execution, followed by automatic zero-level (global) and first-level (linear) correction of the spectral phase. Ultimately, the metabolites for fitting and quantification were selected, with creatine (Cr) employed as a reference to obtain the metabolite ratios of the thalamus, specifically *N*-acetylaspartate (NAA)/Cr, choline (Cho)/Cr, myo-inositol (mI)/Cr, and glutamate and glutamine complex (Glx)/Cr. As illustrated in Figure 2,

### Statistical analyses

The analyses were conducted using the statistical software package SPSS 26.0. In the case of normally distributed measures, these were expressed as mean ± standard deviation ($\bar{X\;}$ ± S). A comparison of the demographic and clinical characteristics of the CSM patients and HCs was conducted using independent samples t-tests, chi-square analysis, and non-parametric tests. A comparative analysis of thalamic metabolites was conducted between CSM patients and HCs, employing the non-parametric independent samples *t*-test. The non-parametric paired-sample t-test were employed to analyze the changes in metabolites in CSM patients during the preoperative and 6-month postoperative periods, respectively. Spearman’s correlation was employed to analyze the changes in ΔmJOA and the differences in thalamic metabolites. A *p*-value of less than 0.05 was considered to indicate a statistically significant difference.

## Results

### Comparison of demographic and clinical characteristics between CSM patients and HCs

The demographic and clinical characteristics of the participants in this study are presented in Table 1 for the reader’s convenience. No significant differences were observed between CSM patients and HCs in terms of age, gender, the presence of diabetes mellitus, the presence of smoking, and BMI.

**Table 1:** A comparison of the demographic and clinical characteristics of CSM patients and HCs.

Clinical features	CSM (*n* = 48)	HCs (*n* = 33)	Statistics (*X^2^*, *t*, *Z*)	*p*-value
Age (years)	54.88 ± 10.84	51.79 ± 12.94	*t* = 1.163	0.248
BMI	23.96 ± 2.24	23.93 ± 2.02	*t* = 0.047	0.963
Sex (male/female)	34/14	18/15	*X^2^* = 2.257	0.133
Diabetes	6	3	*X^2^* = 0.230	0.631
Cigarette smoking	25	12	*X^2^* = 1.947	0.163
Preoperative mJOA score	9.63 ± 2.79	18 ± 0	------	------
mJOA score at 6 months postoperatively	12.56 ± 2.48	18 ± 0	------	------
ΔmJOA	2.94 ± 1.45	0 ± 0	*------*	------

### Comparison of metabolite radios concentrations in CSM patients and HCs

The NAA/Cr, Cho/Cr, mI/Cr, and Glx/Cr ratios measured in the left and right thalamus in the CSM patients were not statistically significant at the preoperative and six-month postoperative time points (Table 2). Furthermore, the metabolite radios measurements taken in the bilateral thalami of the healthy controls were also not statistically significant (Table 3). Accordingly, the mean values of the metabolites in the left and right thalami were used in both studies.

**Table 2:** Comparison of metabolites between the left and right sides of the thalamus in the CSM patients.

	Thalamic metabolites	Right side	Left side	Statistics (*t*, *Z*)	*p*-value
Preoperative	NAA/Cr	1.62 ± 0.14	1.62 ± 0.14	*t* = 0.029	0.977
	Cho/Cr	0.68 ± 0.08	0.67 ± 0.09	*t* = 0.581	0.563
	mI/Cr	0.67 ± 0.12	0.67 ± 0.10	*t* = −0.199	0.843
	Glx/Cr	0.54 ± 0.19	0.54 ± 0.18	*t* = −0.017	0.987
Six months after surgery	NAA/Cr	1.68 ± 0.16	1.70 ± 0.13	*t* = −0.388	0.699
	Cho/Cr	0.72 ± 0.10	0.71 ± 0.09	*t* = 0.639	0.525
	mI/Cr	0.74 ± 0.13	0.74 ± 0.11	*Z* = −0.128	0.898
	Glx/Cr	0.51 ± 0.18	0.51 ± 0.17	*t* = 0.081	0.936

**Table 3:** Comparison of metabolites between the left and right sides of the thalamus in the HCs.

	Thalamic metabolites	Right side	Left side	Statistics (*t*, *Z*)	*p*-value
Preoperative	NAA/Cr	1.75 ± 0.16	1.77 ± 0.14	*Z* = −1.116	0.264
	Cho/Cr	0.78 ± 0.07	0.77 ± 0.07	*t* = 0.445	0.658
	mI/Cr	0.77 ± 0.13	0.78 ± 0.12	*t* = −0.172	0.864
	Glx/Cr	0.55 ± 0.20	0.55 ± 0.12	*t* = −0.053	0.958
Six months after surgery	NAA/Cr	1.79 ± 0.15	1.81 ± 0.15	*t* = −0.543	0.589
	Cho/Cr	0.77 ± 0.08	0.77 ± 0.07	*Z* = −0.115	0.908
	mI/Cr	0.79 ± 0.08	0.78 ± 0.09	*t* = 0.517	0.607
	Glx/Cr	0.54 ± 0.21	0.55 ± 0.18	*Z* = −0.302	0.763

**Figure 3:**
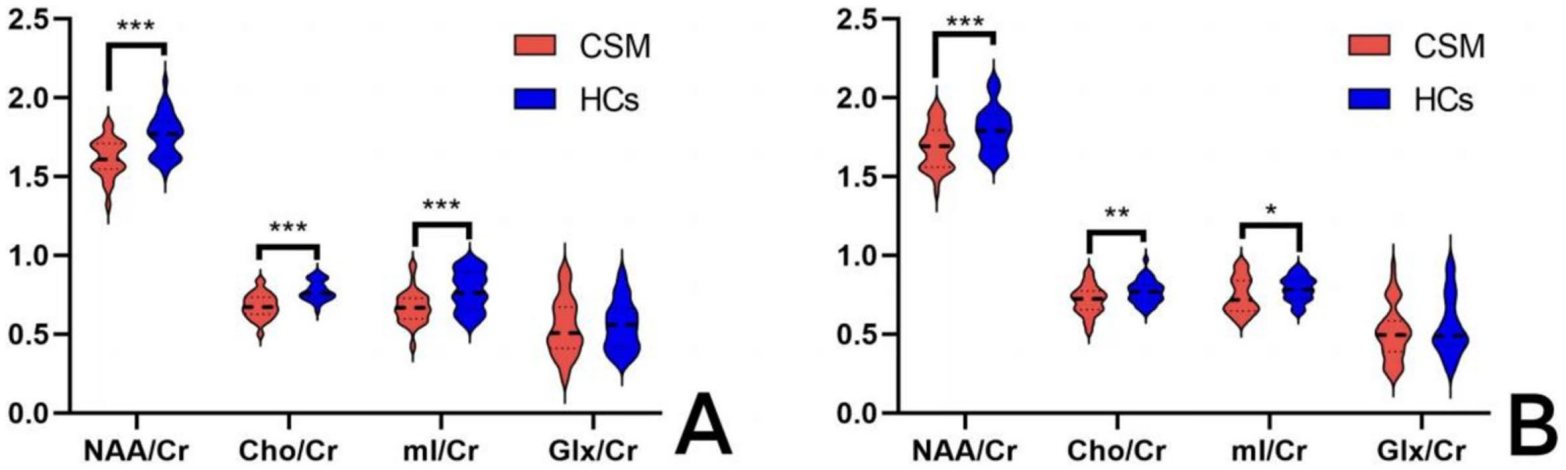
Comparison of thalamic metabolites between the CSM patients and HCs. **(A)** Comparison of thalamic metabolites before surgery; **(B)** Comparison of thalamic metabolites 6 months after surgery. *** Indicates *p* < 0.001; ** indicates *p* < 0.01; * indicates *p* < 0.05.

The values of NAA/Cr, Cho/Cr, and mI/Cr were found to be significantly lower in the thalamus of CSM patients compared to HCs, both prior to surgery and at 6 months postoperatively (*p* < 0.05). In contrast, no significant difference was observed in the values of Glx/Cr in the thalamus of CSM patients compared to HCs (Table 4; Figure 3).

**Table 4:** Comparison of metabolites between CSM patients and HCs.

	Thalamic metabolites	CSM	HCs	*Z*	*p*-value
Preoperative	NAA/Cr	1.62 ± 0.12	1.76 ± 0.14	−4.235	<0.001*
	Cho/Cr	0.68 ± 0.08	0.78 ± 0.06	−5.050	<0.001*
	mI/Cr	0.67 ± 0.11	0.77 ± 0.13	−3.739	0.001*
	Glx/Cr	0.54 ± 0.18	0.55 ± 0.16	−0.293	0.769
Six months after surgery	NAA/Cr	1.69 ± 0.14	1.80 ± 0.14	−3.184	0.001*
	Cho/Cr	0.72 ± 0.09	0.77 ± 0.07	−2.624	0.007*
	mI/Cr	0.74 ± 0.11	0.79 ± 0.08	−2.014	0.044*
	Glx/Cr	0.51 ± 0.17	0.55 ± 0.19	−0.678	0.498

*Indicates statistically significant results.

### Comparison of thalamic metabolite ratios measured at two time points in HCs and CSM patients

No statistically significant differences were observed in the metabolite ratios measured at both time points in the HCs (Table 5).

**Table 5:** Comparison of metabolites between the baseline and the six-month follow-up in HCs.

Thalamic metabolites	Baseline	Six months follow-up	*Z*	*p*-value
NAA/Cr	1.76 ± 0.14	1.80 ± 0.14	−0.929	1.000
Cho/Cr	0.78 ± 0.06	0.77 ± 0.07	0.422	1.000
mI/Cr	0.77 ± 0.13	0.79 ± 0.08	−0.841	1.000
Glx/Cr	0.55 ± 0.16	0.55 ± 0.19	−0.276	0.783

**Figure 4:**
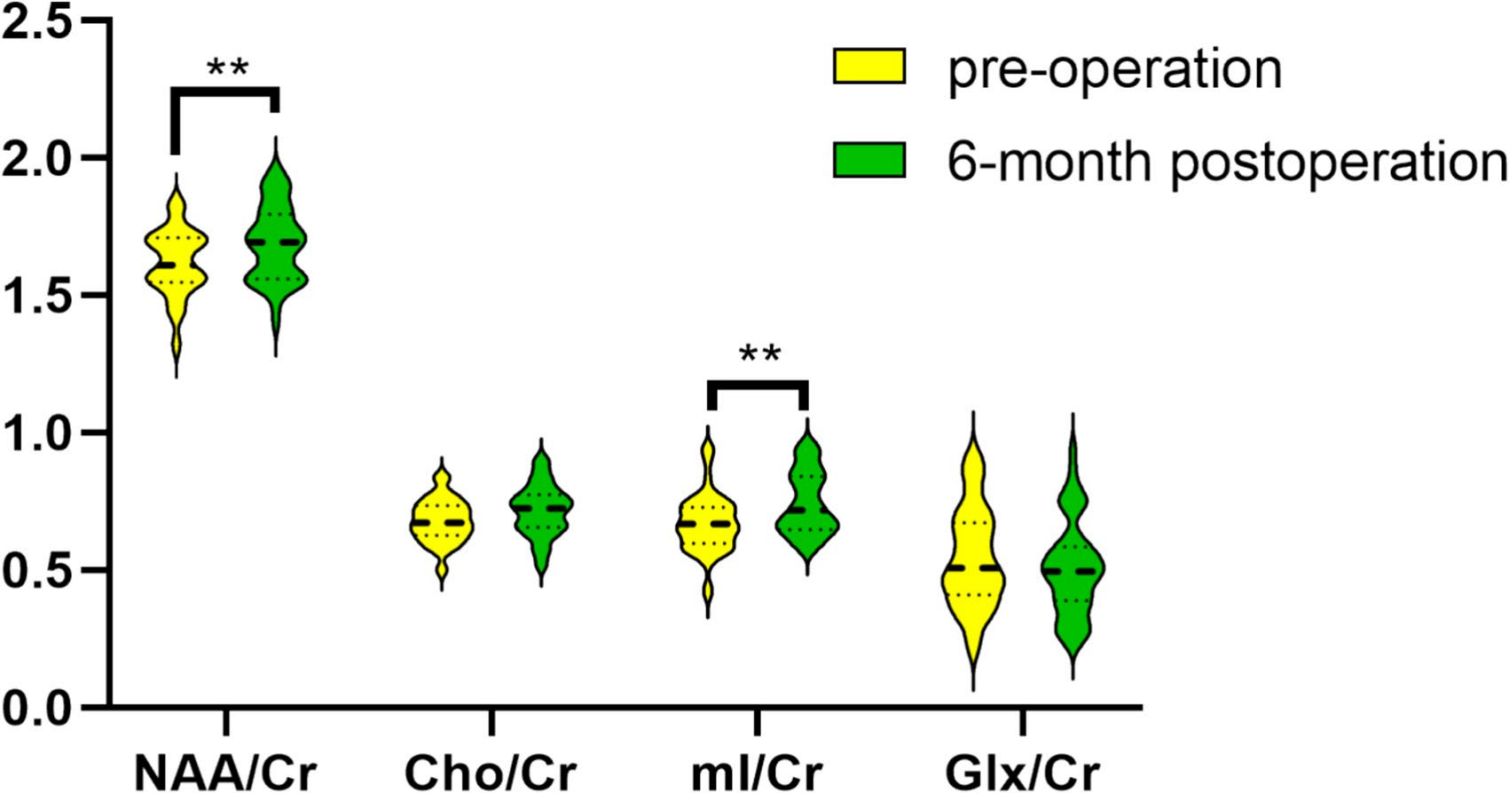
Comparison of metabolites between preoperative and 6-month postoperative periods in the CSM patients. ** Indicates *p* < 0.05.

In comparison to the preoperative period, the concentrations of NAA/Cr and mI/Cr in the thalamus were found to be significantly elevated in CSM patients 6 months following decompression surgery (*p* < 0.05). In contrast, the observed changes in the Cho/Cr and Glx/Cr ratios were not statistically significant, as illustrated in Table 6; Figure 4.

**Table 6:** Comparison of metabolites between preoperative and 6 months postoperative in the CSM patients.

Thalamic metabolites	Preoperative	Six months after surgery	*Z*	*p*-value
NAA/Cr	1.62 ± 0.12	1.69 ± 0.14	−2.285	0.041*
Cho/Cr	0.68 ± 0.08	0.72 ± 0.09	−2.178	0.176
mI/Cr	0.67 ± 0.11	0.74 ± 0.11	−2.925	0.021*
Glx/Cr	0.54 ± 0.18	0.51 ± 0.17	−0.810	0.418

*Indicates statistically significant results.

### Correlation analysis of single voxel changes in thalamic metabolites with ΔmJOA

**Figure 5:**
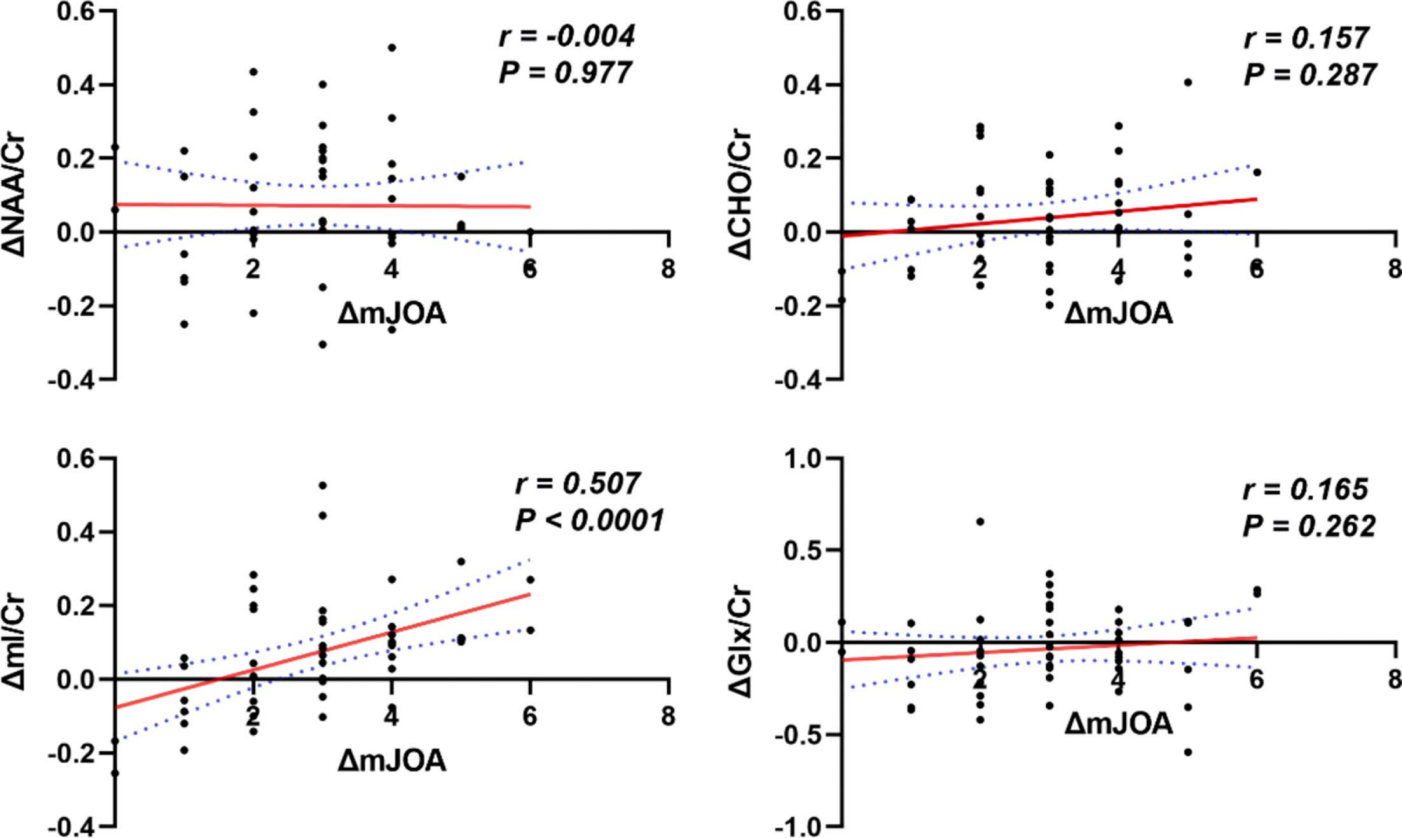
Correlation plots of each metabolite radios and ΔmJOA in CSM patients.

No significant correlation was observed between ΔNAA/Cr, ΔCho/Cr, and ΔGlx/Cr and ΔmJOA in CSM patients. Conversely, a significant correlation was identified between ΔmI/Cr and ΔmJOA (*p* < 0.05), as illustrated in Figure 5.

## Discussion

This study represents the inaugural investigation of metabolites in the thalamus of CSM patients utilizing ^1^H-MRS technology. The metabolites NAA/Cr, Cho/Cr, and mI/Cr were scanned both preoperatively and at the six-month postoperative follow-up. The results revealed a statistically significant reduction in these metabolites in CSM patients relative to healthy controls. Additionally, a noteworthy elevation in NAA/Cr and mI/Cr was observed in comparison to the preoperative phase. Moreover, a correlation was identified between the change in mI/Cr and the change in mJOA in CSM patients during the preoperative and six-month postoperative follow-up periods.

Prior research has demonstrated that CSM not only results in damage to the spinal cord itself, but also gives rise to secondary brain damage. Moreover, the reduction in sensory input from the distal spinal cord to the thalamus following injury to the spinal cord results in the disruption of thalamocortical circuits. This may be a contributing factor to the altered clinical functioning observed in patients with CSM. A voxel-based morphometric (VBM) analysis conducted by Bernabéu-Sanz Á et al. demonstrated a reduction in grey matter volume in the primary sensorimotor cortex and thalamus in patients with CSM, indicating that CSM may result in brain atrophy (11). In a study by PENG X et al. of thalamic functional magnetic resonance imaging (fMRI) in patients with CSM, it was demonstrated that thalamic-cortical functional connectivity is altered both preoperatively and postoperatively. This suggests that adaptive alterations may contribute to the maintenance of cortical sensorimotor networks prior to and following spinal cord decompression (12). In our investigation of the thalamus, we observed a notable decline in thalamic metabolites (NAA/Cr, Cho/Cr, mI/Cr) prior to surgery and at the 6-month postoperative mark in patients with CSM when compared with HCs. This finding provides further evidence in support of the proposed hypothesis.

NAA is an amino acid that is synthesized in the mitochondria of neurons and is present in both the neurons themselves and their axons. It has been demonstrated that NAA is a neurobiological marker that reflects the structural and functional integrity of neuronal mitochondria (19). Consequently, NAA levels can be used as an indicator of the integrity of neuronal mitochondria. It can thus be proposed that NAA content is indicative of the functional status of neurons, with a reduction in NAA content suggestive of neuronal damage. The NAA/Cr ratio in the thalamus of CSM patients was found to be significantly lower than that of the HCs, indicating that secondary damage to thalamic neurons may occur following spinal cord damage in CSM patients. The primary location of Cho is within cell membranes (20), and modifications in its composition predominantly reflect metabolic alterations in cell membranes, which are frequently initiated by ischemic diseases. In light of these findings, it can be posited that the observed decline in Cho/Cr is indicative of a reduction in the number of thalamic neuronal cells. The extensive disruption of cell membranes ultimately results in a diminution of the Cho peak. MI is primarily located within glial cells and plays a role in facilitating neuronal connectivity. In a study conducted by Paquette AF et al. on interface organotypic slices prepared in the hippocampus of neonatal mice in culture, an increase in the number of synapses was observed when mI treatment was added to mature brain tissue. In light of these findings, the observed reduction in mI/Cr in the present study may be indicative of a notable decline in thalamic synapses in CSM patients (21). Glx is a complex of glutamate and glutamine. Glutamate is an excitatory neurotransmitter that participates directly in neurotransmission through the glutamate-glutamine cycle (17). The absence of alterations in Glx/Cr in CSM patients both pre- and post-operatively indicates that glutamate neurotransmission is not disrupted.

Moreover, a comparison of the metabolites of the CSM patients both before and 6 months after surgery revealed a significant increase in NAA/Cr and mI/Cr. The present study demonstrated an increase in NAA/Cr postoperatively, which is inconsistent with the findings of other studies that have examined the preoperative to postoperative changes in NAA/Cr in motor and sensory cortex in patients with CSM, which reported a decrease (4, 22). The rationale behind this analysis may be that the number of cases is relatively limited, which consequently precludes the ability to account for the more overt compensatory changes. This may indicate that the thalamus, as a crucial node within the cortico-basal ganglia thalamic circuit, plays a pivotal role in the communication between the cortex and subcortical regions. Furthermore, it suggests that neurons within the thalamus are given priority for recovery during neural restoration. MI is primarily located within astrocytes, which are responsible for maintaining the internal and external environment of neuronal cells. Furthermore, astrocytes play a role in the survival, migration, immune modulation, signaling and axonal growth of neurons. Moreover, after decompression, there exist fibrous lateral bud growth in the injured spinal cord (23), and the new synapses of sensorimotor cortex was established or the original inhibitory synapse was removed (24), so the sensory afferent information was restored to normal, and the transmission function of spinal cord-thalamus tract was recovered. An increase in mI/Cr is indicative of an enhancement in the trophic function of neuronal cells, and, in a macroscopic view, a recuperation of thalamic neurological function. It has been put forth that mI is elevated during the initial phases of CSM and subsequently returns to baseline levels over time (25). The findings of the present study provide support for this hypothesis. The Cho/Cr ratio exhibited no discernible change between the preoperative and postoperative periods, suggesting that the metabolic function of neuronal cell membranes had not yet recovered to an adequate extent by the six-month postoperative mark.

The mJOA score is a widely used method for assessing neurological function in patients with CSM. A reduction in the mJOA score indicates the presence of varying degrees of neurological impairment in patients with CSM (26, 27). The present study employed a comparison of the mJOA scores, which revealed a decline in neurological function in the CSM patients relative to the HCs. The present study yielded a significant positive correlation between thalamic ΔmI/Cr and ΔmJOA in CSM patients, representing a novel finding, to the best of our knowledge. Prior research has indicated that the concentration of mI is elevated in the white matter of individuals diagnosed with multiple sclerosis (28). A previous ^1^H-MRS study demonstrated that the level of mI was elevated in patients with moderate to severe head injury. This increase was attributed to astrocytosis (15, 29). The precise function of MI in the central nervous system (CNS) remains unclear. However, it is known to be preferentially concentrated in glial cells. One potential function of MI in the CNS is that of an osmotic agent (30). Moreover, research has demonstrated that mI concentrations in the precentral gyrus are markedly elevated in CSM patients with a relatively brief duration of symptoms (≤6 months) (25). This phenomenon may be attributed to the proliferation of reactive glial cells, which occurs in conjunction with subacute neuroinflammation (31). It has been demonstrated that glial scarring, which is formed by the overproliferation of astrocytes in the late stages of spinal cord injury, has a significant negative impact on neuronal axonal regeneration (32). Tsitsopoulos PP et al. demonstrated that the number of astrocytes was elevated in the spinal cord of CSM patients, and established a correlation between this finding and the observed neurological dysfunction (33). Ibrahim et al. observed the presence of astrocyte proliferation in the vicinity of chronic compression spinal cord injury in rats (34). Astrocytes play a pivotal role in providing support within the nervous system (35). The present study demonstrated that the ascending recovery of mI/Cr in CSM patients after surgery was consistent with the pathological manifestations of mI. Furthermore, the correlation between the findings ΔmI/Cr and ΔmJOA indicated that postoperative neurological recovery in CSM patients is reversible.

The present study is limited by three factors. The sample size is relatively modest, which may limit the generalizability of the findings. Secondly, the ^1^H-MRS examination was conducted manually, which introduces the possibility of discrepancies between the preoperative and postoperative voxels. Finally, the mJOA score is not the best scale for measuring sensory function. It is therefore recommended that subsequent studies adopt a more specialized research methodology.

## Conclusion

In conclusion, the ^1^H-MRS examination represents a valuable tool for the detection of changes in the levels of relevant metabolites in the brain following chronic spinal cord injury in CSM patients. It is possible that the thalamic ΔmI/Cr may be associated with the recovery of sensory nerves, which could serve as a potential clinically referable index.

## Data Availability

The original contributions presented in the study are included in the article/supplementary material, further inquiries can be directed to the corresponding authors.
